# Effect of Spirulina Intervention on Oxidative Stress, Antioxidant Status, and Lipid Profile in Chronic Obstructive Pulmonary Disease Patients

**DOI:** 10.1155/2015/486120

**Published:** 2015-01-22

**Authors:** Md. Ismail, Md. Faruk Hossain, Arifur Rahman Tanu, Hossain Uddin Shekhar

**Affiliations:** Department of Biochemistry and Molecular Biology, University of Dhaka, Dhaka 1000, Bangladesh

## Abstract

*Background and Objective.* Oxidative stress is intimately associated with many diseases, including chronic obstructive pulmonary disease (COPD). Study objectives include a comparison of the oxidative stress, antioxidant status, and lipid profile between COPD patients and controls and evaluation of the effect of spirulina intervention on oxidative stress, antioxidant status, and lipid profile of COPD patients. *Methods.* 30 patients with COPD and 20 controls with no respiratory problems were selected. Global Initiative for Chronic Obstructive Lung Disease criteria were served as the basis of COPD diagnosis. The serum content of malondialdehyde (MDA), lipid hydroperoxide, glutathione (GSH), vitamin C, cholesterol, triglyceride (TG), and high density lipoprotein (HDL) was measured. The activity of superoxide dismutase (SOD), catalase (CAT), and glutathione-s-transferase (GST) was also measured. Two different doses, (500 × 2) mg and (500 × 4) mg spirulina, were given to two groups, each of which comprises 15 COPD patients. *Results.* All targeted blood parameters have significant difference (*P* = 0.000) between COPD patients and controls except triglyceride (TG). Spirulina intake for 30 and 60 days at (500 × 2) mg dose has significantly reduced serum content of MDA, lipid hydroperoxide, and cholesterol (*P* = 0.000) while increasing GSH, Vit C level (*P* = 0.000), and the activity of SOD (*P* = 0.000) and GST (*P* = 0.038). At the same time, spirulina intake for 30 and 60 days at (500 × 4) mg dose has favorable significant effect (*P* = 0.000) on all targeted blood parameters except for HDL (*P* = 0.163).

## 1. Introduction

The terminology “oxidative stress” points a shift in the equilibrium between oxidants and antioxidants in favor of oxidants. From neurological disorders [1, 2] to cancer [3] and asthma [4, 5], the contribution of oxidative stress is well documented. Cigarette smoke holds many oxidants, organic compounds, and free radicals. The components of tobacco smoke spark off the inflammation cascade which acts as a double edged sword. The lung has heavy reliance on superoxide dismutase (SOD) (EC 1.15.1.1), catalase (CAT) (EC 1.11.1.6), and glutathione peroxidase (GSH-Px) (EC 1.11.1.9) as they are the major enzymatic antioxidants of the lungs. Dismutation of superoxide to hydrogen peroxide (H_2_O_2_) and oxygen (O_2_) by SOD is of rudimentary importance for every cell as superoxide is the primary reactive oxygen species (ROS) produced from a variety of sources. Human lung expresses entire three forms of superoxide dismutase, that is, CuZn-SOD, Mn-SOD, and EC-SOD, to a great degree [6, 7]. CAT and GSH-Px generate water (H_2_O) and O_2_ by reducing H_2_O_2_ that is engendered by the action of SOD or the action of oxidases, such as xanthine oxidase. CAT exists as a tetramer consisting of 4 identical monomers, each of which contains a heme group at the active site [8]. The enzyme responsible for the reduction of H_2_O_2_ and lipid hydroperoxides is glutathione peroxidase, which contains the unique amino acid selenocysteine within the active sites and uses low-molecular-weight thioles, such as GSH, to reduce H_2_O_2_ and lipid peroxides to their corresponding alcohols [9]. Glutathione-S-transferase (GST) (EC 2.5.1.18), another antioxidant enzyme family, inactivates secondary metabolites, such as unsaturated aldehydes, epoxides, and hydroperoxides [10]. Vitamin C reduces oxidative stress and improves vascular function and structure. These effects may be mediated via modulation of enzyme systems that generate free radicals [11, 12]. Glutathione (GSH) provides antioxidant defenses by their ability to exist in reversible oxidized and reduced forms [13, 14]. A low level of GSH in the cells increases its risk of the oxidative damage. It has a role in converting vitamins C and E back to their active forms. GSH protects cells against apoptosis by interacting with proapoptotic and antiapoptotic signaling pathways [15]. Reactive oxygen species are highly short lived and therefore the best way to estimate oxidative stress is to quantify the products of their reaction with lipids, proteins, and nucleic acids. Thiobarbituric acid reactive substances measure is one such test which quantifies malondialdehyde (MDA), a product of lipid peroxidation [16]. Spirulina is a microscopic and a filamentous cyanobacterium (blue-green alga) that has a long history of use as a food for humans. Spirulina is a rich source of protein and vitamins, especially vitamin B12, minerals, carotenoids, and phycocyanins [17]. Spirulina has received much attention as functional food regarding its association with cholesterol regulatory properties and antioxidant capacity [18]. Conjunction therapy with bronchodilators and anti-inflammatory drugs has helped to improve the pulmonary function in patients, as proven by a significant increase in FEV_1_% and FEV_1_/FVC% over a period of 4 months, but the patients reported to be experiencing certain side effects such as palpitation, weakness, and tremors on the use of these medications. The patients, those receiving medication + spirulina, showed a significant improvement in the FVC% (72 ± 17 to 78 ± 16) and FEV_1_% (61 ± 14 to 67 ± 16) over a period of 4 months. Further, the role of spirulina gets prominent when a significant decrease in FVC%, FEV_1_%, and FEV_1_/FVC% was observed in the next 2 months when the spirulina supplements were withdrawn while the medication was continued [19]. The six-minute walking distance (6MWT) test is a commonly used test to estimate functional exercise capacity in patients with chronic diseases including chronic obstructive lung disease [20]. FEV_1_ (forced expired volume in one second) is the volume expired in the first second of maximal expiration after a maximal inspiration and is a useful measure of how quickly full lungs can be emptied. FEV_1_/FVC is the FEV_1_ expressed as a percentage of the FVC (whichever volume is larger) and gives a clinically useful index of airflow limitation [21] (see Table 2). The interaction between oxidants and antioxidants and their association with the aetiology of COPD is well supported by several studies [22, 23]. Alteration in lung functions during the follow-up period was not examined. This study has concentrated on the changes in blood parameters as a result of spirulina intervention. This study was designed with the purpose of comparing oxidative stress, antioxidant status, and lipid profile between COPD patients and controls (healthy individuals) in the context of our own population and at the same time evaluating the effect of spirulina intervention in COPD patients, whether spirulina intervention can bring in some changes in the targeted blood parameters and to what extent, at 2 different doses of spirulina.

## 2. Methods

### 2.1. Subjects

A total of 30 COPD patients (divided into 2 groups: 15 patients consumed 500 mg spirulina 2 times/day and another 15 patients consumed 1000 mg spirulina 2 times/day) and 20 controls (all males) were selected as the study population. Stable COPD patients were administered with spirulina together with their usual medication for two months. Controls were not treated with spirulina. Spirulina (1 g/day) for a period of 2 months was administered in the enrolled asthma patients (from Shri Sayajirao General Hospital, Vadodara) suffering from mild to moderate degree of bronchial asthma [19]. In our study, we provided 2 g/day dose together with 1 g/day dose in order to check whether the increase in spirulina consumption could make more favorable changes. Baseline data of oxidative stress, antioxidant status, and lipid profile were established experimentally so that changes in the measured blood parameters are solely for the intervention of spirulina. Their usual medication did not exert any contradictory effect on this study. Subjects were asked to keep usual diet and prohibited to take any functional foods or dietary supplements. The compliance of all subjects was confirmed by telephone twice a week. Patients with COPD were included if their forced expiratory volume in 1 s (FEV_1_)/forced vital capacity ratio was <70%. Spirometric tests were performed while the patients were in stable condition using Spirobank G MIR 0476. Individuals with a history suggestive of bronchial asthma, bronchiectasis, cystic fibrosis, and fibrosis of pulmonary tuberculosis were excluded. All patients were recruited from National Institute of Chest Diseases and Hospital (NIDCH), Mohakhali, Dhaka. Individuals with no respiratory problems strictly with an FEV_1_/forced vital capacity ratio > 80% were selected as controls. Subjects were excluded from the control group if they reported difficulty in breathing while working or walking at any point of time in their life, had or had had exposure to risk factors other than smoking, ceased to smoke at any point of time in their life due to breathing problems, or visited any physician due to respiratory problems. Insufficiencies in executing spirometry decently were counterbalanced by these criteria.

### 2.2. Sampling and Data Collection

As per standard procedures, anthropometric mensurations like height and weight were collected. In cohort with the World Health Organization recommendations, [24] subjects were classified into different categories of body mass index. An integrated questionnaire was employed in order to obtain information on age, education, occupation, and smoking status. The data and blood samples were collected over a period of 4 months (from February 2014 to May 2014). Under sterile conditions, about 5 mL of blood was collected into EDTA vacutainers through venipuncture. The samples were centrifuged at 1000 g for 15 minutes to separate plasma.

### 2.3. Preparation of Spirulina Capsules

After purchasing spirulina from a commercial company (Dinajpur Agro BD limited), it was exposed to gamma radiation (6 Gy) at IRPT (Institute of Radiation and Polymer Technology), in the Bangladesh Atomic Energy Commission, Bangladesh, to make it decontaminate from bacteria and other pathogens. In an automated way, spirulina was incorporated into capsule shells. And finally spirulina capsules were incorporated into containers under an aseptic condition through the use of laminar hood with proper precautions.

### 2.4. Biochemical Assays

CAT activity was assessed by the method of Góth [25]. Activity of the GST was determined by using 1-chloro-2,4-dinitrobenzene [26]. The activity of SOD was assessed by the method of Beyer Jr. and Fridovich [27]. GSH was estimated by the method of Beutler et al. [28] using 5,5′-dithiobis-(2-nitrobenzoic acid). Vit C was estimated by the method of Lowry et al. [29]. MDA was measured as thiobarbituric acid reactive substances in plasma [30]. Lipid hydroperoxide was estimated by the method of Yagi [31]. Cholesterol, triglyceride (TG), and high density lipoprotein (HDL) were measured by using cholesterol, TG, HDL measuring reagent (Vitro Scient, Egypt). All reagents used were of analytical grade and obtained from sigma chemicals, Germany.

### 2.5. Statistical Analysis

Statistical analysis was carried out using SPSS 16.0 version, and *P* values were set at 0.05. Independent *t* test was performed to explore the statistically significant difference between COPD patients and controls. Data are presented as mean ± SD (standard deviation). One-way analysis of variance was performed to assess the mean values.

An account of the nature of the work was given to the patients and informed consent was obtained, providing that they hold the rights to withdraw themselves from the study at any time they like. The study protocol was approved by the Human Ethics Committee of Dhaka University.

## 3. Results

Demographic characteristics of COPD patients and controls are illustrated in Table 1. The age of the study subjects in the year ranged from 45 to 60. The majority of the study population were >50 years old. There were more COPD patients with body mass index < 18.5 kg/m (13.33%) compared with controls (5%). Half of the patients fell into the class of heavy tobacco users (50%). All of the patients (100%) abstained from smoking upon their diagnosis of COPD.

Comparative serum content of oxidants (MDA, lipid hydroperoxide), antioxidants (Vit C, GSH), cholesterol, TG, and HDL together with the activity of burst enzymes (CAT, SOD, and GST) between COPD patients and controls at day 0 (i.e., on the set of experiment) is presented in Table 3. Effect of spirulina intervention at (500 × 2) mg, (500 × 4) mg doses on the serum content of oxidants, antioxidants, cholesterol, TG, and HDL and on the activity of burst enzymes in COPD patients is presented in Tables 4 and 5, respectively.

## 4. Discussion

The outcomes of the present investigation have affirmed the presence of an oxidant-antioxidant imbalance in COPD patients when compared with healthy subjects (controls). Patients have high levels of MDA and low levels of antioxidants in comparison with controls. High levels of MDA in COPD patients have been one of the most consistent findings across several studies [32–34]. In comparison with the controls, COPD patients have decreased serum content of GSH, Vit C and reduced serum activity of CAT, SOD, and GST. Similar enzymatic (SOD, CAT, and GST) and nonenzymatic (GSH, Vit C) antioxidant imbalance between COPD patients and controls have been described by Arja et al. [16]. The results of the present analysis clearly highlight the efficacy of spirulina as an MDA lowering agent as spirulina intervention, which for 30 days at (500 × 2) mg and (500 × 4) mg doses has resulted in 12.17% and 17.72% reduction in MDA level, respectively, and for 60 days at (500 × 2) mg and (500 × 4) mg doses has resulted in 20.89% and 35.71% reduction in MDA level, respectively (see Table 6). These outcomes are similar to the finding of Lu et al. 2006 [35]. The inhibitory effects of spirulina on lipid peroxidation are in accordance with the findings of Lee et al. [17]. With reference to antioxidant enzymes, namely, SOD, CAT, GST, and GSH, they were increased significantly after intake of spirulina. Supplementation with spirulina for 30 days at (500 × 2) mg and (500 × 4) mg doses has resulted in 4.98% and 11.08% increase in SOD level, respectively, and for 60 days at (500 × 2) mg and (500 × 4) mg doses has resulted in 12.84% and 20.19% increase in SOD level, respectively. Lu et al. [35] have described an increase in the activity of superoxide dismutase in their study relating to spirulina intervention. Increase in catalase activity is only significant at (500 × 4) mg dose for 30 and 60 days. Many animal and human studies have repeatedly reported the lipid-lowering effects of spirulina. Park and Kim [36] reported that the plasma levels of triglyceride, total cholesterol, and LDL-cholesterol in Korean elderly people were significantly decreased after spirulina supplementation. Also, spirulina supplementation to type 2 diabetic patients resulted in a significant decrease in plasma concentrations of triglyceride, total cholesterol, and LDL-cholesterol (Iyer et al.) [37]. The findings of our study regarding cholesterol and triglyceride reducing effect of spirulina supplementation are in accordance with the findings of Park and Kim [36] as well as Iyer et al. [37]. Supplementation with spirulina for 30 days at (500 × 2) mg and (500 × 4) mg doses has resulted in 1.48% and 3.25% reduction in TG levels, respectively, and for 60 days at (500 × 2) mg and (500 × 4) mg doses has resulted in 2.85% and 6.24% reduction in TG levels, respectively. Reduction in TG level is significant only at (500 × 4) mg dose for 30 and 60 days. Spirulina intervention for 30 days at (500 × 2) mg and (500 × 4) mg doses has resulted in 3.03% and 7.14% reduction in cholesterol level, respectively, and for 60 days at (500 × 2) mg and (500 × 4) mg doses has resulted in 9.05% and 15.84% reduction in cholesterol level, respectively. Reduction in cholesterol level is significant at both (500 × 2) mg and (500 × 4) mg doses for 30 and 60 days. Lee et al. [17] reported high density lipoprotein (HDL) increasing effect of spirulina intervention. In accordance with their study, spirulina supplementation in this study for 30 days at (500 × 2) mg and (500 × 4) mg doses has resulted in 1.2% and 2.26% increase in HDL level, respectively, and for 60 days at (500 × 2) mg and (500 × 4) mg doses has resulted in 2.94% and 5.88% increase in HDL level, respectively. But an increase in HDL level at (500 × 2) mg and (500 × 4) mg doses for 30 and 60 days is nonsignificant. Supplementation with spirulina for 30 days at (500 × 2) mg and (500 × 4) mg doses has resulted in 9.21% and 11.84% increase in the Vit C level, respectively, and for 60 days at (500 × 2) mg and (500 × 4) mg doses has resulted in 21.05% and 28.94% increase in the Vit C level, respectively. Vit C increase could be attributed to spirulinas good content of Vit C [38]. Intervention with spirulina for 30 days at (500 × 2) mg and (500 × 4) mg doses has resulted in 8.23% and 13.42% increase in GSH level, respectively, and for 60 days at (500 × 2) mg and (500 × 4) mg doses has resulted in 19.08% and 27.67% increase in GSH level, respectively. Various hypotheses have been proposed in an attempt to identify direct mechanisms responsible for the hypolipidemic and hypocholesterolemic potency of Spirulina. High gamma-linolenic acid (GLA) content of spirulina has gained the attention. Prostaglandin (PG) synthesis in our body requires GLA as a precursor. Regulation of a variety of basic biochemical functions in the body including the regulation of cholesterol synthesis requires the prostaglandin PGE1. An external food source of GLA such as spirulina, therefore, plays a crucial role in regulating the cholesterol levels [19]. In conclusion, spirulina intervention brought favorable effect against oxidative stress. Also, we have observed favorable effects of spirulina intervention on blood lipids and antioxidant status, in our targeted patients with chronic obstructive pulmonary disease (COPD). Our results also suggest that spirulina is a promising agent as a functional food for the management of COPD. Further studies with larger sample size and longer duration are required to ascertain the mechanism of spirulina actions on lipid profiles, oxidative stress, and antioxidant status.

## 5. Conclusion

The present study has concluded with the outcomes of having an imbalance in oxidative stress, antioxidant status, and lipid profile between COPD patients and controls. Moreover, antioxidant status and lipid profile of COPD patients are proven to be improved through an interventional 2 months course of spirulina. Oxidative stress is shown to be reduced as a result of spirulina intervention.

## Figures and Tables

**Table 1:** Demographic features of COPD patients and controls.

	COPD (*n* = 30)		Controls (*n* = 20)	
	*n*	%	*n*	%
Age				
45–50	08	26.66	10	50
50–55	09	30	07	35
55–60	13	43.33	03	15
BMI				
16–18.5	04	13.33	01	05
18.5–25	26	86.67	19	95
Smoking category				
≤10 (light)	04	13.33	04	20
11–20 (moderate)	11	36.66	01	05
21–30 (heavy)	15	50	00	00

BMI: body mass index; COPD: chronic obstructive pulmonary disease.

**Table 2:** Clinical features of COPD patients and controls.

	COPD		Control		*t* value	*P* value
	Mean	SD	Mean	SD		
FEV_1_/FVC (%)	54.47	6.62	95.1	8.91	−20.048	0.000
6MWT (ft)	515.4	127.15	803.33	99.16	−9.7805	0.000

FEV_1_: forced expiratory volume in 1 s; FVC: forced vital capacity; 6MWT: 6-minute walk test.

**Table 3:** Comparative serum content of oxidants, antioxidants, Cholesterol, TG, and HDL together with the activity of burst enzymes between COPD patients and controls at day 0 (i.e., on set of experiments).

Parameters	COPD (*n* = 30) (mean ± SD)	Controls (*n* = 20) (mean ± SD)	*t* value	*P* value
Malondialdehyde (MDA) (nmoL/mL)	3.78 ± 0.24	1.2 ± 0.19	45.623	0.000^*^
Lipid hydroperoxide (nmoL/mL)	3.63 ± 0.19	1.25 ± 0.17	50.752	0.000^*^
Catalase (kU/L)	31.53 ± 8.41	59.88 ± 7.04	−14.156	0.000^*^
Superoxide dismutase (SOD) (U/mL)	67.25 ± 6.43	104.33 ± 11.18	−15.746	0.000^*^
Glutathione-S-transferase (GST) (U/mL)	28.41 ± 4.02	39.14 ± 3.34	−11.225	0.000^*^
Glutathione (GSH) (nmoL/mL)	279.3 ± 23.26	414.67 ± 20.32	−24.006	0.000^*^
Vitamin C (mg/dL)	0.76 ± 0.07	2.15 ± 0.16	−42.646	0.000^*^
Cholesterol (mg/dL)	167.22 ± 10.33	188.05 ± 13.21	−6.805	0.000^*^
Triglyceride (TG) (mg/dL)	172.10 ± 14.44	179.67 ± 18.66	−1.757	0.084
High density lipoprotein (HDL) (mg/dL)	42.21 ± 5.17	51.95 ± 6.36	−6.513	0.000^*^

^*^Statistically significant.

**Table 4:** Effect of spirulina intervention at (500 × 2) mg dose on serum content of oxidants and antioxidants and on the activity of burst enzymes in COPD patients.

Parameters	0 days (mean ± SD)	30 days (mean ± SD)	60 days (mean ± SD)	*F* value	*P* value
Malondialdehyde (MDA) (nmoL/mL)	3.78 ± 0.24	3.32 ± 0.26	2.99 ± 0.22	85.083	0.000^*^
Lipid hydroperoxide (nmoL/mL)	3.63 ± 0.19	3.41 ± 0.21	2.79 ± 0.22	136.057	0.000^*^
Catalase (kU/L)	31.53 ± 8.41	33.33 ± 8.30	34.05 ± 8.08	0.740	0.480
Superoxide dismutase (SOD) (U/mL)	67.25 ± 6.43	70.64 ± 6.20	75.89 ± 5.34	15.742	0.000^*^
Glutathione-S-transferase (GST) (U/mL)	28.41 ± 4.02	29.14 ± 4.05	30.95 ± 3.50	3.408	0.038^*^
Glutathione (GSH) (nmoL/mL)	279.3 ± 23.26	302.28 ± 18.42	332.61 ± 13.61	60.399	0.000^*^
Vitamin C (mg/dL)	0.76 ± 0.07	0.83 ± 0.06	0.92 ± 0.05	52.971	0.000^*^
Cholesterol (mg/dL)	167.22 ± 10.33	162.15 ± 9.03	152.08 ± 7.95	21.233	0.000^*^
Triglyceride (TG) (mg/dL)	172.10 ± 14.44	169.55 ± 14.15	167.18 ± 12.22	0.975	0.381
High density lipoprotein (HDL) (mg/dL)	42.21 ± 5.17	42.72 ± 5.12	43.45 ± 5.20	0.436	0.648

^*^Statistically significant. Comparisons are made between day 0 versus day 30 and day 0 versus day 60.

**Table 5:** Effect of spirulina intervention at (500 × 4) mg dose on serum content of oxidants and antioxidants and on the activity of burst enzymes in COPD patients.

Parameters	0 days (mean ± SD)	30 days (mean ± SD)	60 days (mean ± SD)	*F* value	*P* value
Malondialdehyde (MDA) (nmoL/mL)	3.78 ± 0.24	3.11 ± 0.25	2.43 ± 0.28	216.404	0.000^*^
Lipid hydroperoxide (nmoL/mL)	3.63 ± 0.19	3.10 ± 0.23	2.44 ± .21	240.034	0.000^*^
Catalase (kU/L)	31.53 ± 8.41	36.12 ± 8.21	42.25 ± 7.59	13.314	0.000^*^
Superoxide dismutase (SOD) (U/mL)	67.25 ± 6.43	74.76 ± 5.64	80.83 ± 4.89	42.864	0.000^*^
Glutathione-S-transferase (GST) (U/mL)	28.41 ± 4.02	31.61 ± 3.75	32.88 ± 3.42	11.350	0.000^*^
Glutathione (GSH) (nmoL/mL)	279.3 ± 23.26	316.76 ± 16.68	356.57 ± 11.71	140.565	0.000^*^
Vitamin C (mg/dL)	0.76 ± 0.07	0.85 ± .06	0.98 ± .05	114.403	0.000^*^
Cholesterol (mg/dL)	167.22 ± 10.33	155.28 ± 7.45	140.72 ± 14.90	41.244	0.000^*^
Triglyceride (TG) (mg/dL)	172.10 ± 14.44	166.50 ± 13.25	161.34 ± 11.68	5.011	0.000^*^
High density lipoprotein (HDL) (mg/dL)	42.21 ± 5.17	43.16 ± 5.15	44.69 ± 4.79	1.853	0.163

^*^Statistically significant. Comparisons are made between day 0 versus day 30 and day 0 versus day 60.

**Table 6:** Comparison between the effects of the 2 doses of spirulina on serum content of oxidants and antioxidants and on the activity of burst enzymes in COPD patients.

Parameters	After 30 days		After 60 days	
	Change in percentage (%) at (500 × 2) mg dose	Change in percentage (%) at (500 × 4) mg dose	Change in percentage (%) at (500 × 2) mg dose	Change in percentage (%) at (500 × 4) mg dose
Malondialdehyde (MDA) (nmoL/mL)	12.17	17.72	20.89	35.71
Lipid hydroperoxide (nmoL/mL)	6.06	14.6	23.14	32.78
Catalase (kU/L)	5.71	14.6	7.93	33.97
Superoxide dismutase (SOD) (U/mL)	4.98	11.08	12.84	20.19
Glutathione-S-transferase (GST) (U/mL)	2.57	11.26	8.94	15.73
Glutathione (GSH) (nmoL/mL)	8.23	13.42	19.08	27.67
Vitamin C (mg/dL)	9.21	11.84	21.05	28.94
Cholesterol (mg/dL)	3.03	7.14	9.05	15.84
Triglyceride (TG) (mg/dL)	1.48	3.25	2.85	6.24
High density lipoprotein (HDL) (mg/dL)	1.2	2.26	2.94	5.88

## Abbreviations

CAT: Catalase
COPD: Chronic obstructive pulmonary disease
FEV_1_: Forced expiratory volume in 1 s
GOLD: Global Initiative for Chronic Obstructive Lung Disease
GPx: Glutathione peroxidase
GSH: Glutathione
GST: Glutathione-s-transferase
MDA: Malondialdehyde
SOD: Superoxide dismutase
HDL: High density lipoprotein
TG: Triglyceride
FVC: Forced vital capacity
6MWT: 6-minute walk test
Vit C: Vitamin C.
